# TRPM7 transactivates the FOSL1 gene through STAT3 and enhances glioma stemness

**DOI:** 10.1007/s00018-023-04921-6

**Published:** 2023-08-29

**Authors:** Shanchun Guo, Vanajothi Ramar, Alyssa A. Guo, Talib Saafir, Hannah Akpobiyeri, Breanna Hudson, Jason Li, Mingli Liu

**Affiliations:** 1 Department of Chemistry, Xavier University, 1 Drexel Dr, New Orleans, LA USA; 2 Department of Microbiology, Biochemistry and Immunology, Morehouse School of Medicine, Atlanta, USA; 3 University of South Carolina SOM Greenville, Greenville, SC USA; 4 Wake Forest University School of Medicine, 475 Vine Street, Winston-Salem, NC USA

**Keywords:** Glioma, TRPM7, FOSL1, STAT3, Glioma stem cells, Signaling pathways

## Abstract

**Introduction:**

We previously reported that TRPM7 regulates glioma cells’ stemness through STAT3. In addition, we demonstrated that FOSL1 is a response gene for TRPM7, and the FOSL1 gene serves as an oncogene to promote glioma proliferation and invasion.

**Methods:**

In the present study, we determined the effects of FOSL1 on glioma stem cell (GSC) markers CD133 and ALDH1 by flow cytometry, and the maintenance of stem cell activity by extreme limiting dilution assays (ELDA). To further gain insight into the mechanism by which TRPM7 activates transcription of the FOSL1 gene to contribute to glioma stemness, we constructed a FOSL1 promoter and its GAS mutants followed by luciferase reporter assays and ChIP-qPCR in a glioma cell line and glioma patient-derived xenoline. We further examined GSC markers ALDH1 and TRPM7 as well as FOSL1 by immunohistochemistry staining (IHC) in brain tissue microarray (TMA) of glioma patients.

**Results:**

We revealed that FOSL1 knockdown reduces the expression of GSC markers CD133 and ALDH1, and FOSL1 is required to maintain stem cell activity in glioma cells. The experiments also showed that mutations of − 328 to − 336 and − 378 to − 386 GAS elements markedly reduced FOSL1 promoter activity. Constitutively active STAT3 increased while dominant-negative STAT3 decreased FOSL1 promoter activity. Furthermore, overexpression of TRPM7 enhanced while silencing of TRPM7 reduced FOSL1 promoter activity. ChIP-qPCR assays revealed that STAT3, present in nuclear lysates of glioma cells stimulated by constitutively activated STAT3, can bind to two GAS elements, respectively. We demonstrated that deacetylation of FOSL1 at the Lys-116 residue located within its DNA binding domain led to an increase in FOSL1 transcriptional activity. We found that the expression of TRPM7, ALDH1, and FOSL1 protein is associated with grades of malignant glioma, and TRPM7 protein expression correlates to the expression of ALDH1 and FOSL1 in glioma patients.

**Conclusions:**

These combined results demonstrated that TRPM7 induced FOSL1 transcriptional activation, which is mediated by the action of STAT3, a mechanism shown to be important in glioma stemness. These results indicated that FOSL1, similar to GSC markers ALDH1 and TRPM7, is a diagnostic marker and potential drug target for glioma patients.

**Supplementary Information:**

The online version contains supplementary material available at 10.1007/s00018-023-04921-6.

## Introduction

Glioblastoma (GBM) is the most aggressive brain cancer in adults. Increasing evidence suggests that glioma stem cells (GSCs) are responsible for glioma initiation, development, and intratumoral heterogeneity. Intratumoral heterogeneity is partially responsible for the high resistance of GBM to conventional treatments [1]. As such, it is essential to identify effective therapeutic targets for eliminating GSCs to treat the tumor and prevent its reccurence. Our previous work demonstrated that TRPM7 induces gliomagenesis and glioma stemness to promote glioma cell survival and invasion, where one of the mechanisms is through regulating its downstream Notch 1/Survivin pathway [2, 3]. However, clinical trials that aim at the Notch pathway showed that the Notch signaling inhibitor, γ-secretase inhibitor (RO4929097), had little clinical efficacy as a single agent in GBM [4, 5], although it indeed produced inhibitory activity of Notch signaling in tumor cells and inhibited CD133^+^ neurosphere formation [5]. The failure of γ-secretase inhibitor in treatment of GBM is partly due to upregulation of key mesenchymal genes and an increase in VEGF-dependent angiogenic factors [5]. Furthermore, the growth and maintenance of GBM are not solely dependent on the Notch pathway. Therefore, effective treatments targeting Notch may require combinations of therapeutic modalities [4].

We reported previously that TRPM7 is an indirect regulator of the FOSL1 gene where high expression of FOSL1 elevates glioma cell proliferation and invasion; FOSL1 is also associated with poor survival in GBM patients [6]. The studies from other groups also support the oncogenic role of FOSL1 in GBM. They found that FOSL1 promotes the proneural to mesenchymal transition of GSCs and is predominantly expressed in the mesenchymal subtype of GSCs [7]. FOSL1 facilitates UBC9-dependent CYLD sumoylation, induces K63-mediated polyubiquitination of major nuclear factor κB (NF-κB) intermediates, and subsequently activates the NF-κB axis [7]. FOSL1 is a key factor in regulating mesenchymal glioblastoma plasticity and contributes to GBM aggressiveness [8]. Therefore, it prompts us further to investigate the mechanism of TRPM7’s regulation of FOSL1. We then analyzed putative transcription factor binding sites on the FOSL1 gene promoter. Interestingly, we found potential signal transducer and activator of transcription 3 (STAT3) binding sites in the promoter. Based on our previous findings that TRPM7 enhances glioma stemness by triggering STAT3 activation [2], in the present study, we will investigate the mechanisms by which TRPM7/STAT3/FOSL1 regulatory axis drives stemness and growth in glioma.

## Materials and methods

### Antibodies and reagents

The following primary antibodies were used in the present study: Rabbit polyclonal anti-TRPM7 (cat. no. ab23455) was purchased from Abcam. Rabbit polyclonal anti-β-actin antibody (cat. no. A3854) was purchased from Sigma-Aldrich; Merck KGaA. Rabbit polyclonal anti-phosphorylated STAT3 (Y705) antibody (cat. no. 9131) was purchased from Cell Signaling Technology, Inc. Rabbit polyclonal PE anti-human CD133 antibody (cat no. 372804) and PE mouse IgG1, κ isotype ctrl (FC) antibody were purchased from BioLegend. Rabbit polyclonal anti-aldehyde dehydrogenase 1 antibody (ALDH1; cat no. GTX123973) was purchased from GeneTex, Inc. Mouse monoclonal anti-FOSL1 antibody (cat. no. sc-283107) was purchased from Santa Cruz Biotechnology, Inc for Western blotting, and rabbit polyclonal anti-FOSL1 antibody was purchased from Sigma-Aldrich (cat. no. SAB2108461) for Immunohistochemistry (IHC) staining. All secondary antibodies (goat anti-rabbit, peroxidase-conjugated, cat. no. AP132P; and goat anti-mouse antibody, peroxidase-conjugated, cat. no. AP124P) used for Western blotting were purchased from Calbiochem; Merck KGaA. STAT3 inhibitor XIII, C188-9, was purchased from EMD Millipore (cat. no. 573128). SR11302 was purchased from TOCRIS (cat. no. 2476).

### Plasmids and siRNA

The wild-type human TRPM7 (wtTRPM7) was provided by Dr. Carsten Schmitz, University of Colorado, Denver, CO. Control scrambled siRNA (On-TARGETplus Non-targeting siRNA, cat. no. D-001810-01-05), ON-TARGETplus SMARTpool siRNA (Cat. no. L-005393-000005) targeting TRPM7, and ON-TARGETplus SMARTpool siRNA (Cat. no.L-003544-00-0005) targeting STAT3 were purchased from Dharmacon (Lafayette, CO). Control scrambled siRNA and siRNA targeting FOSL1 (siRNA ID # s15585) were purchased from Thermo Fisher Scientific (Waltham, MA). The scrambled siRNAs, with no homology to any known sequences, were used as controls. FOSL1 human shRNA lentivirus particles (sh FOSL1 lentiviral particles) were purchased from Origene (TL312944V, Rockville, MD). These shRNA constructs were designed against multiple splice variants at the gene locus and contained four unique 29mer target-specific shRNA and one scramble control. FOSL1 human GFP-tagged ORF clone (RG202104) was purchased from Ogigene. The STAT3 constructs, including constitutively active (STAT3-CA), dominant negative STAT3 (STAT3-DN), and control vector, were provided by Dr. Jacqueline F. Bromberg and described previously [9].

*Construction of site-specific mutants of the FOSL1 promoter, FOSL1 mutants at the DNA binding domain, and Luciferase Reporter Assays* (1) DNA was extracted from HTB-138 cells using the Blood & Cell Culture DNA Mini Kit (Qiagen) according to the manufacturer’s instructions. The 5′-flanking region of the human FOSL1 gene was amplified from HTB-138 DNA and cloned into a pGL3-Basic vector (Promega, cat. no. E1751) between MluI [New England Biolabs Inc (NEB), Ipswitch, MA, NEB, cat. no. R0198L] and BglII (NEB, cat. no. R0144L) sites. (2) Primers for amplification (human FOSL1 Luc or hFOSL1 Luc) are listed in Table 1. The site-specific mutants that disrupted STAT3 binding sites (− 328 to − 336 FOSL1 Luc mut1, − 378 to − 386 FOSL1 Luc mut2, and the double mutant containing both binding sites) were created by a PCR-based approach using Q5 site-directed mutagenesis kit (NEB, cat. no. E0554S). The primers used to make the mutations in the binding sites (hFOSL1 Luc mut 1 and hFOSL1 Luc mut 2) are shown in Table 1. (3) The FOSL1 mutants at the DNA binding domain that contains FOSL1 acetylated lysine residue (K116), including the deacetylation mimic K116R mutant, and three acetylation mimic mutants, K116A, K116E, and K116Q, were generated by a PCR-based approach using Q5 site-directed mutagenesis kit with a FOSL1 human GFP-tagged ORF clone as a template. Primers for amplification are listed in Table 1. 4) For the reporter assays, 5 × 10^4^ cells were seeded in a 24-well plate and transfected with either wild type (FOSL1 Luc) or a variety of mutants of FOSL1, along with TRPM7 construct, or siRNA TRPM7 (siTRPM7), or transduced with various STAT3 constructs into glioma cells for 24–72 h as needed. The protein lysates were made using a Dual-Luciferase Reporter (DLR) Assay system (Promega, cat. no. E1910). Firefly and *Renilla* luciferase activities were then measured using a DLR Assay system. The firefly luminescence was normalized to *Renilla* luminescence as an internal control for transfection efficiency. Experiments were performed three times.

**Table 1:** List of primers used in the study

Primer set	Forward 5′–3′	Reverse 5′–3′
TRPM7	CTTTGACCAAGAGGGAATGTG	GACCAAGCGACCACAAAAAC
FOSL1	CTCCAGGGGTACGTCGAAG	TCAGTTCCTTCCTCCGGTTC
GAPDH	GAAGGTGAAGGTCGGAGTC	GAAGATGGTGATGGGATTTC
hFOSL1 Luc	ATCGACGCGTCAACCTGTGCCAGCTACTCA	ATCGAGATCTATGAAAAGTTCTCGGGCTGA
hFOSL1 Luc mut 1	CCACCATTTTTGTCGCCCTGGTTTGTCATTT	CATATTTTATTTTACTTTATATTTTTGAGACCG
hFOSL1 Luc mut 2	GAAGCCTTAAAACCGGGCCAGTGGAAAGACCT	GAGTATATGAAAAGTGAATAAATGACAAACCAG
hFOSL1 ChIP for mut 1	GGAAGTTGAGCCTGCAGTG	AGTGAATAAATGACAAACCAGGG
hFOSL1 ChIP for mut 2	ACCCTGGTTTGTCATTTATTCAC	CCGTTTCTGCTCCCACAAAA
K116R	CGCGAGCGGAACCGGCTGGCTGCGGCC	CCTTACTCGGCGGCGCTCCTCTTCCTC
K116A	CGCGAGCGGAACGCGCTGGCTGCGGCC	CCTTACTCGGCGGCGCTCCTCTTCCTC
K116E	CGCGAGCGGAACGAGCTGGCTGCGGCC	CCTTACTCGGCGGCGCTCCTCTTCCTC
K116Q	CGCGAGCGGAACCAGCTGGCTGCGGCC	CCTTACTCGGCGGCGCTCCTCTTCCTC

### Cell culture

Human glioblastoma cell lines: A172 (RRID: CVCL_0131) and U87MG (HTB-14), a glioblastoma of unknown origin (RRID: CVCL_0022) were obtained from American Type Culture Collection (ATCC). All cells were cultured in Dulbecco’s modified Eagle’s medium (DMEM) supplemented with 10% fetal bovine serum (FBS; both from Thermo Fisher Scientific, Inc.), 50 units/ml penicillin, and 50 µg/ml streptomycin at 37 °C. Patient derived xenoline (PDX)-tumor tissue cubes stored at liquid nitrogen were provided by Dr. Yancey G. Gillespie at the University of Alabama at Birmingham (UAB). PDX-L14 line was generated by implanting PDX-tumor tissue cubes subcutaneously into the flanks of male or female 6–8 weeks old nude mice under anesthesia (ketamine/Xylazin 90/6 mg/kg BW). Briefly, cryopreserved tumor tissues were thawed at 37 °C and washed with phosphate-buffered saline (PBS) before subcutaneous implantation. To prepare a single-cell suspension of viable tumor cells, the xenograft tumor tissues were harvested and minced with scalpel blades followed by passes through cell strainers. The cells were then grown in DMEM/F-12 media plus 10% FBS, 50 units/ml penicillin, and 50 µg/ml streptomycin for future use. All experiments were performed with mycoplasma-free cells. PDX-L14 cells with proneural subtype have wild-type genes including EGFR, PTEN, CDKN2A, NF-κB, CDK4/MDM2, and amplified gene of CSNK2A, deleted TP53, and expression of CD133.

*Enrichment for glioma stem cells* We enriched the GSCs by growing neurospheroid cultures as described previously [2]. Briefly, glioma cells cultured in conventional tissue culture media were grown to confluence and dissociated using 0.1% trypsin and dispersed by pipetting with a 23-gauge needle. After checking for single cells, the cells were pelleted and suspended in sphere enrichment medium, specifically, human neurobasal medium supplemented with B27, 20 ng/ml EGF and 20 ng/ml FGF-2 (Invitrogen, Carlsbad, CA, USA), and 5 µg/ml heparin (Sigma-Aldrich, St. Louis, MO). These cells were then plated in ultra-low attachment surface tissue culture plates (Corning). Following overnight incubation at 37 °C with 5% CO_2_, the distinct non-adherent human GSCs were apparent in culture. These spheres were collected, gently centrifuged at low speed (1000 rpm), and passaged and maintained for growth in the sphere enrichment medium for future use.

### Transfection of siRNA and DNA constructs

When glioma cells grew to reach about 50–75% confluency, the appropriate amount of specific siRNAs and corresponding controls with a final concentration of 100 nM were transfected using Lipofectamine RNAiMAX reagent in serum-free OptiMEM-1 medium (Invitrogen, Carlsbad, CA) according to manufacturer's instruction. 48 or 72 h post-transfection, target knockdowns were assessed by quantitative real-time RT-PCR (qRT-PCR) analysis or Western blot, accordingly. The glioma spheres enriched from U87MG or PDX-L14 were infected with sh FOSL1 lentiviral particles by lipofectamine 3000 transfection reagent (Invitrogen, Carlsbad, CA) according to the manufacturer's instruction. The knockdown efficiency of FOSL1 were measured 72 h post-infection by Western blot.

### Extreme limiting dilution assays (ELDA)

The glioma spheres enriched from U87MG or PDX-L14 were infected with sh FOSL1 lentiviral particles by lipofectamine 3000 transfection reagent, then decreased cell densities were plated in ultra-low attachment 96-well plates with fresh medium added every 3 days. The number of positive wells for the presence of spheres was counted 2 weeks after plating. The glioma spheres enriched from U87MG or PDX-L14 were also treated with 10 µM SR11302 for 6 h, and then decreased cell densities were plated in ultra-low attachment 96-well plates with fresh medium supplemented with SR11302 being added every 3 days. Limiting dilution analysis was performed using ELDA R package (http://bioinf.wehi.edu.au/software/elda/) [10].

### Flow cytometry

To evaluate CD133 expression by flow cytometry, cells were harvested, washed with Cell Staining Buffer (cat. no. 420201; Biolegend, Inc.), and then incubated with PE anti-human CD133 antibody (1:200; cat. no. 372803; Biolegend, Inc.) for 15–20 min on ice in the dark. Cells were then washed and suspended in Cell Staining Buffer (at room temperature for 5 min) for analysis. The data acquired on the Guava easyCyte 8HT Base System were analyzed using the InCyte software. ALDH1 enzymatic activity was assessed using an Aldefluor kit (cat. no. 01700; STEMCELL Technologies Inc.) according to the manufacturer’s instructions. Cells suspended in the Aldefluor assay buffer were incubated with ALDH enzyme substrate, BODIPY-aminoacetaldehyde (BAAA; 1:200) for 30–60 min at 37 °C. As a control for baseline fluorescence, cells were also treated for 30–60 min at 37 °C with the ALDH inhibitor, diethylaminobenzaldehyde (DEAB at 1:100 dilution). Fluorescence was detected using the Guava easyCyte 8HT Base System and analyzed using the InCyte software. Statistical significance was determined by the paired Student’s t test or one-way ANOVA test.

### Quantitative real-time RT-PCR (qRT-PCR)

Total RNA isolation, cDNA synthesis, and PCR amplification were performed as previously described [11]. Total RNA was isolated from cells using a RNeasy Kit (Qiagen, Valencia, CA, USA) and quantified using the Nanodrop N-1000 by Agilent Biosystems (Santa Clara, CA). Purified total RNA (0.75 μg) was reverse transcribed using the iScript cDNA Synthesis Kit (Bio-Rad Laboratories, Inc, Hercules, CA, USA) according to manufacturer’s protocol. Reverse transcription was performed by using random hexamers at 25 °C for 5 min, 42 °C for 30 min, and 85 °C for 5 min. After diluting ten times, the cDNA was then amplified using iQ SYBR Green Supermix (Bio-Rad Laboratories, Inc.) according to manufacturer’s protocol under the following conditions: activation of the Taq DNA polymerase at 95 °C for 3 min, 40 cycles at 95 °C for 10 s (denaturation), and 61 °C for 45 s (combined annealing and extension). The quantitative gene analysis utilized the CFX Connect Real-Time PCR Detection System. Each condition was conducted in biological triplicates, and each biological replicate was amplified in technical triplicates. Relative expression for each gene was evaluated using the 2^−ΔΔCt^ Livak method, and GAPDH was used as the reference gene [11].

### Western blotting

Cells were lysed with lysis buffer of M-PER™ Mammalian Protein Extraction Reagent (Thermo Fisher Scientific cat. no. 78501) supplemented with Halt™ Protease and Phosphatase Inhibitor Single-Use Cocktail (100x) (Thermo Fisher Scientific cat. no. 78442). SDS/PAGE separated samples, and separated proteins were transferred to nitrocellulose membranes and identified by immunoblotting. Primary antibodies were obtained from commercial sources and were diluted to a ratio of 1:500 or 1:1000 according to manufacturer's instruction. Blots were developed with Supersignal Pico or Femto substrate (Pierce). Densitometric analysis of the bands was performed with the ImageQuant program (Bio-Rad).

### Immunofluorescence staining

The glioma cells were fixed in 4% PFA, permeabilized with 0.02% Triton-X-100 (MilliporSigma), and blocked with 10% goat serum. The cultures were then incubated with a primary antibody, diluted in the blocking buffer in a 1:100 ratio, overnight at 4 °C, followed by incubation with the FITC or Cy3 conjugated secondary antibody for 1 h at room temperature. Finally, the cultures were washed with PBS and mounted with VECTASHIELD mounting medium with DAPI. The samples were imaged using a Zeiss AxioImager, the upright epifluorescence microscope, or a Zeiss Confocal Microscope LSM700, AxioObserver equipped with a camera. Total nuclear immunofluorescence intensity with TRPM7 and phosphorylated STAT3 (pSTAT3) antibodies were determined with an open source project Fiji software.

### Tissue microarray (TMA)

Glioma tissue arrays from glioma patients were purchased from BioCoreUSA Corporation (https://biocoreusa.com/default.aspx). Biopsy features included age, sex, organ or anatomic site involved, grading, and pathological diagnosis (HE-stained sections). Slides (product no. GL1001b) contained 75 cases of glioma: grade II, n = 51 (astrocytoma, n = 47; oligodendroglioma, n = 2; oligoastrocytoma, n = 2); grade III, n = 12 (anaplastic astrocytoma); grade IV, n = 12 (glioblastoma), and 10 cases of normal brain tissues.

### Immunohistochemistry (IHC)

IHC staining was performed on 5-µm thick microarray slides. The slides were baked at 55–60 °C for 30 to 60 min and pretreated in citrate buffer of pH 6 for 10 min at 100 °C. The slides were then incubated with different primary antibodies diluted at 1:100 at 4 °C overnight. Briefly, immunohistochemical staining was performed using the rabbit polyclonal anti-TRPM7, rabbit polyclonal anti-ALDH1, and rabbit polyclonal anti-FOSL1 antibody, which is specific for TRPM7, ALDH1 and FOSL1, respectively. The secondary antibody was diluted at 1:200 and incubated at room temperature for 60 min. A streptavidin–biotin unlabeled immunoperoxidase technique (ABC-Elite; cat. no. PK-6101, Vector Laboratories, Inc.) with diaminobenzidine (DAB) [DAB Substrate Kit, Peroxidase (HRP), cat. no. SK-4100, Vector Laboratories, Inc.] was used as a chromogen. Mayer’s hematoxylin was used for nuclear counterstaining for 2 min. The slides were then visualized under the light microscope.

*HSORE determination.* The staining intensity of cells in TMA was evaluated as negative or positive in three different bright fields (≥ 100 cells/field). The semi-quantitative HSCORE was calculated for different antigens such as TRPM7, ALDH1, and FOSL1, using the following equation: HSCORE = Ʃpi (i + 1), where ‘i’ is the intensity with a value of 0, 1, 2, or 3 (negative, weak, moderate, or strong, respectively), and ‘pi’ is the percentage of stained cells for each intensity [12, 13]. Immunohistochemically stained slides were blindly reviewed and scored by two independent investigators.

### ChIP-qPCR analysis

The chromatin immunoprecipitation (ChIP) coupled with quantitative PCR (qPCR) assay was performed using the Pierce Agarose ChIP Kit (Thermo Scientific, cat. no. 26156). The glioma cells as indicated were crosslinked with 1% formadehyde and inactivated by 125 mM glycine. Samples then undergone Micrococcal Nulease digestion (MNase Digestion) generating chromatin fragments (0.2–1 kb), which were then incubated with 5 μg of polyclonal anti-phosphorylated STAT3 or normal rabbit IgG (negative control) on a rocker overnight at 4 °C. Then, protein A/G agarose beads were added, and the chromatin was incubated for 1 h at room temperature. Antibody-bound protein/DNA complexes were eluted, reverse-crosslinked by incubation with proteinase K at 65 °C for 40 min, and subjected to qPCR. An aliquot of chromatin that was not incubated with an antibody was used as the input control sample (10% total input sample). qPCR was performed using primers designed to surround the − 328 to − 336 and − 378 to − 386 binding sites of the FOSL1 promoter using the Primer 3 tool (https://primer3.ut.ee/). The primer sequences for each site (hFOSL1 ChIP for mut 1 and hFOSL1 ChIP for mut 2) were listed in Table 1. Quantitative PCR was performed using Bio-Rad SYBR QPCR Master Mix (Bio-Rad, cat. no. 1708882) with the CFX Connect Real-Time PCR Detection System (Bio-Rad). Data were analyzed using the percent input method and using normal IgG as a negative control as in Reference [14].

### Statistical analysis

The results obtained in the present study are expressed as the mean ± SD of at least 3 independent experiments conducted in triplicate. GraphPad Prism 9 (GraphPad Software, Inc.) was used for statistical analysis. Paired Student’s t test or one-way ANOVA followed by Holm-Sidak post hoc tests were performed for data analysis, and p < 0.05 was considered to indicate a statistically significant difference.

## Results

### FOSL1 downregulation suppresses glioma stemness and tumor growth

**Fig. 1:**
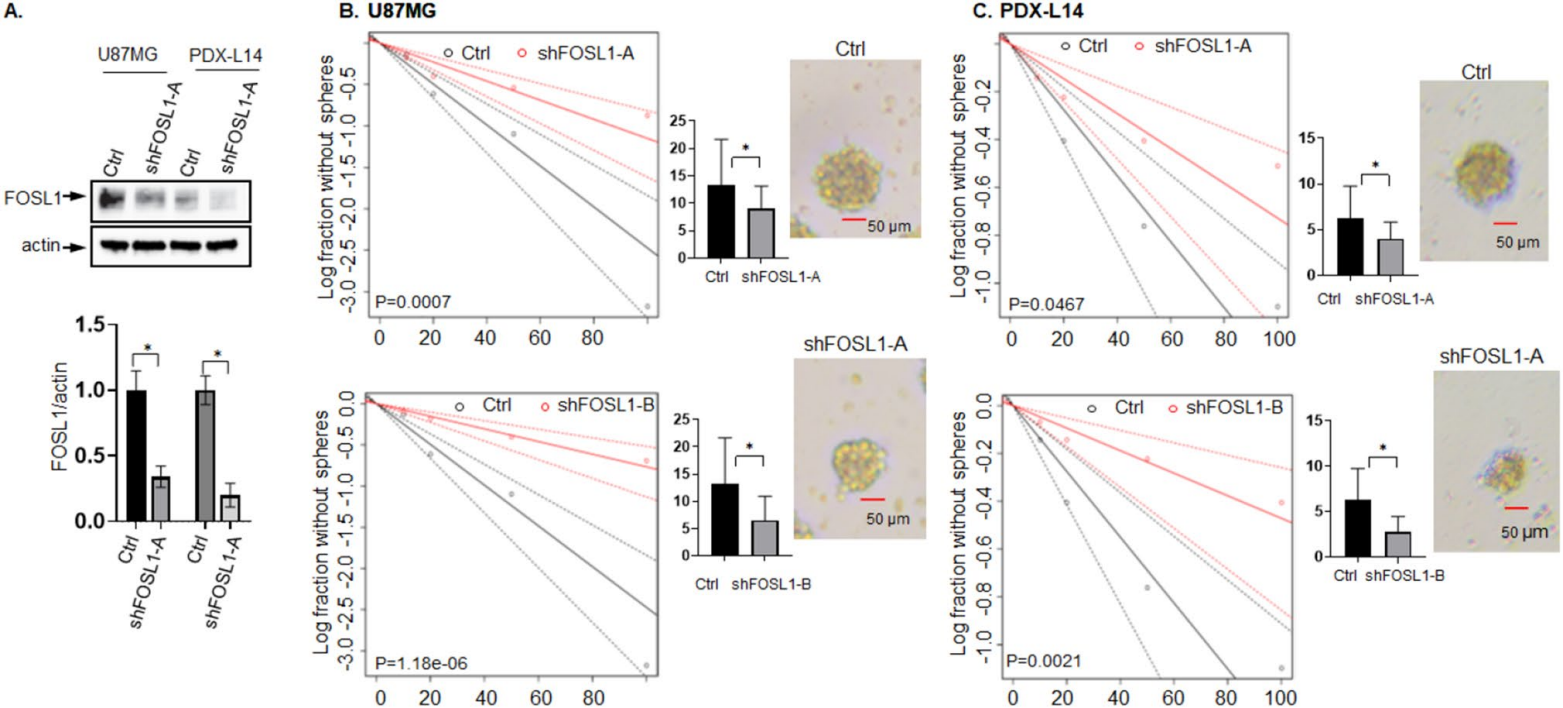
FOSL1 knock-down impairs stemness in vitro. **A** Significant silencing efficiencies were confirmed in both GSCs enriched from U87MG and PDX-L14 cells. **B** Representative limiting dilution experiments on U87MG GSCs infected with shFOSL1-A, shFOSL1-B, and controls. Bar plot shows the estimated stem cell frequency with the confidence interval; chi-square p < 0.001. **C** Representative limiting dilution experiments on PDX-L14 GSCs infected with shFOSL1-A, shFOSL1-B, and controls. Bar plot shows the estimated stem cell frequency with the confidence interval; chi-square p < 0.05

We previously reported that cell viability was significantly decreased when FOSL1 was silenced by siRNA in glioma cell lines as compared to controls [6]. Considering that glioma heterogeneity, attributable in part to the presence of GSCs activity, is an important aspect that contributes to glioblastoma aggressiveness, we examined the effects of FOSL1 on glioma cell stemness by measuring stem cell frequencies. To accomplish the aims of this goal, we cultured glioma cells U87MG and PDX-L14 as gliospheres in a serum-free medium that enriches GSCs, and then silenced FOSL1 expression using lentiviral delivery of shRNA FOSL1, followed by extreme limiting dilution assays (ELDA). As shown in Fig. 1A, high silencing efficiencies were confirmed at protein levels in both cell types (Fig. 1A). FOSL1 silencing decreased the sphere-forming capacity of U87MG-derived GSCs with an estimated stem cell frequency of shFOSL1-A = 86.9 compared to control ctrl = 40.5 (chi-square p = 0.000711); while shFOSL1-B = 130.5 compared to control ctrl = 40.5 (chi-square p = 1.18e−06, Fig. 1B). Similarly, silencing of FOSL1 decreased the sphere-forming ability of patient-derived xenoline (PDX-L14)-derived GSCs with an estimated stem cell frequency of shFOSL1-A = 212.1 compared to control ctrl = 72.6 (chi-square p = 0.00215); while shFOSL1-B = 137.4 compared to control ctrl = 72.6 (chi-square p = 0.0467, Fig. 1C). FOSL1, one of the members of the FOS family, is a subunit of the transcriptional complex activator protein (AP-1). To get more evidence to strengthen the conclusion, we used SR11302 to specifically inhibit AP-1/FOSL1 activity and followed by an ELDA assay. As shown in Supplement Fig. 1A, treatment with SR11302 at 10 µM for 6 h significantly reduced the FOSL1 protein expression. SR11302 reduced the sphere-forming ability of U87MG-derived GSC with an estimated stem cell frequency of SR11302 = 126.3 compared to control ctrl = 49.5 (chi-square p = 0.0001, Suppl 1B). Similar to U87MG cells, SR11302 decreased the sphere-forming potential of PDX-L14-derived GSC with an estimated stem cell frequency SR11302 = 145.4 compared to control ctrl = 55.5 (chi-square = 0.002, Suppl 1C). These data indicated that FOSL1 inhibitor has effects on compromising GSC stemness. Together, these results showed that FOSL1 is required for the maintenance of stem cell activity in glioma cells. The data from A172 was not included because we failed to generate the tumor spheres from A172 cells after multiple attempts.

### FOSL1 knockdown reduces the expression of GSC markers CD133 and ALDH1

**Fig. 2:**
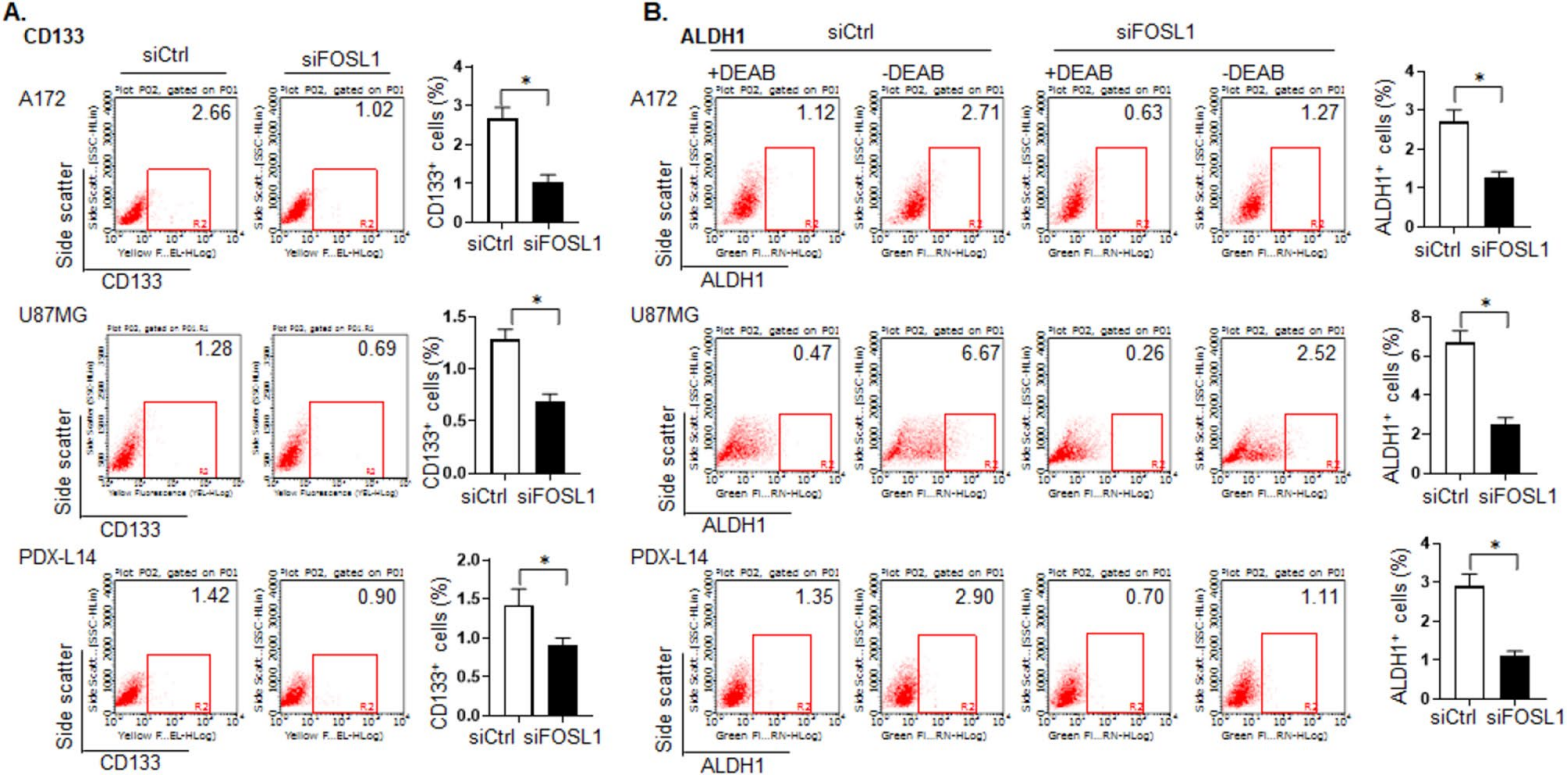
**A** Flow cytometric analysis to measure CD 133 expression was performed in A172, U87MG, and PDX-L14 glioma cells. **B** ALDH1 enzymatic activities determined by the Aldefluor assay was performed in A172, U87MG, and PDX-L14 glioma cells

Since FOSL1 is essential for maintenance GSC activity, we hypothesized that it would regulate GSC marker expression levels in glioma cells. We therefore investigated whether FOSL1 affects the GSC markers CD133 and ALDH1 upon silencing FOSL1 gene in A172, U87MG, and PDX-L14 cells. We found that when FOSL1 was knocked down by siRNA FOSL1 (siFOSL1), the number of CD133^+^ cells decreased from 2.66 to 1.02% in A172, from 1.28 to 0.69% in U87MG, and from 1.42 to 0.90% in PDX-L14 cells, respectively (Fig. 2A). To further verify our findings, ALDH1, another GSC marker, was assayed by ALDEFLUOR. FOSL1 silencing decreased the number of ALDH1-positive cells from 2.71 to 1.27% in A172, from 6.67 to 2.52%, and from 2.90 to 1.11% in PDX-L14 (Fig. 2B). These results indicated that the downregulation of FOSL1 led to a reduction in GSC population. Diethylaminobenzaldehyde (DEAB) is a specific ALDH inhibitor used to control background fluorescence.

### TRPM7 induced FOSL1 transcriptional activation in gliomagenesis is mediated by STAT3

#### Site-specific mutations of the GAS motifs of the FOSL1 promoter markedly reduces promoter activity in glioma cells

**Fig. 3:**
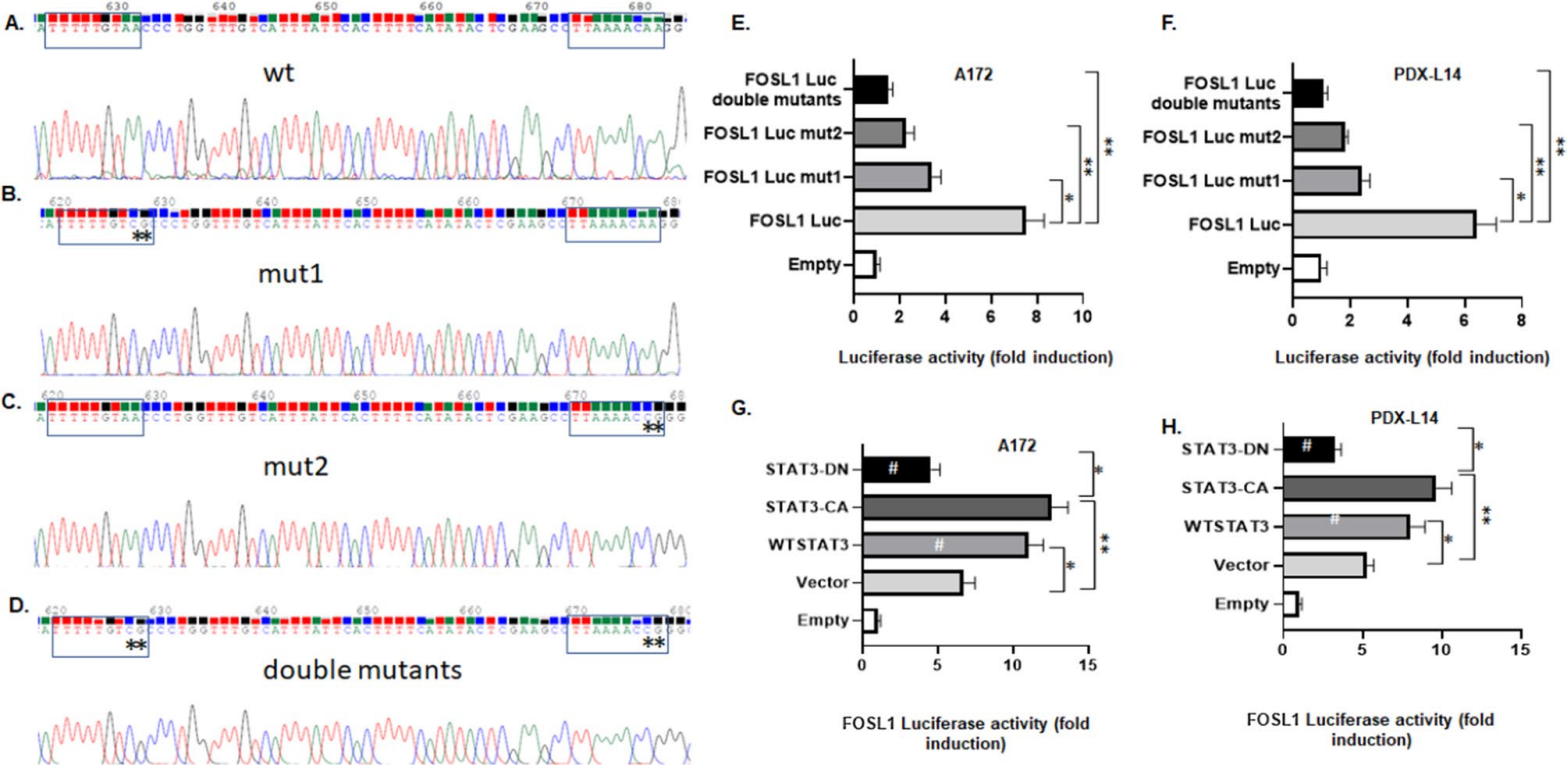
Site-specific mutation of the GAS motifs of the FOSL1 promoter markedly reduces promoter activity, while transduction of STAT3 enhances FOSL1 promoter activity in glioma cells. **A**–**D** Primers hybridizing to the most 5′ extent of the − 1000 FOSL1 promoter sequence construct were used to create the FOSL1 promoter (**A**) and its STAT3 mutants (**B**, **C**), in which two consecutive STAT3 consensus sites were eliminated by making a two-base change in each site. A double STAT3 mutant was also created in which both the GAS motifs were mutated (**D**). For each mutant construct, the mutated bases are depicted in stars. **E**, **F** These constructs were transiently transfected into A172 (**E**) and PDX-L14 (**F**) cells. Following a 24 h incubation period, cells were lysed and analyzed for luciferase activity. The relative expression refers to the fold changes in luciferase activity over the vector alone. Values expressed are the average of three independent experiments plus or minus the standard deviation of the mean. **G**, **H** Either wild-type STAT3 (WTSTAT3), a constitutively active mutant of STAT3 (STAT3-CA), or a dominantly negative mutant STAT3 (STAT3-DN) was cotransfected with the FOSL1 promoter construct into A172 (**G**) and PDX-L14 (**H**) cells. Following a 72 h incubation period, cells were lysed and analyzed for luciferase activity as described as in **E**, **F**

We previously demonstrated that FOSL1 is a response gene for TRPM7 and downregulation of FOSL1 can hinder glioma proliferation and invasion [6]. TRPM7 regulates glioma stemness through STAT3 activation [2]. To gain insight into the regulation of TRPM7-induced transcription of FOSL1, we then analyzed the sequences of FOSL1 promoter (FOSL1 Luc) in the − 1000 to 200 regions for putative transcription factor binding sites. The GAS elements is of the sequence TT(N5)AA, and it is known that STAT3 is capable of binding to this consensus sequence [15]. We found two GAS elements are located at positions − 328 to − 336 and − 378 to − 386 of the FOSL1 promoter region. To point to the important roles for the GAS elements in activating FOSL1 transcription, we constructed both FOSL1 promoter and its mutants in which a few critical bases in the binding sites of STAT3 were mutated. As shown in Fig. 3, we used FOSL1 Luc construct as a template (Fig. 3A, wt) to make constructs with site-specific mutations in either the − 328 to − 336 [Fig. 3B (mut 1 or mut 1 STAT3)] or the − 378 to − 386 [Fig. 3C (mut 2 or mut 2 STAT3)] of the GAS elements. We also made constructs in which both the − 328 to − 336 and − 378 to − 386 GAS elements were mutated (double mutants, or double mutant STAT3, Fig. 3D). The mut 1 construct contains two base changes that disrupts the − 328 to − 336 GAS element in which the adenines at − 328 and − 329 were changed to cytidine and guanine, whereas mut 2 constructs disrupts − 378 and − 386 GAS element in which the adenines at − 378 and − 379 GAS element were changed to cytidine and guanine. Finally, the double mutant contains both the mutations present in the mut 1 and mut 2. Each construct was subcloned into the pGL3-Basic luciferase reporter vector and the promoter/reporter constructs were transiently transfected into A172 or PDX-L14 cells, and luciferase reporter assays were performed. The results of these assays are shown in Fig. 3E, F. The mut 1 STAT3 mutation resulted in a 2.2-fold decline in luciferase activity in A172 cells, while the mut 2 STAT3 mutation was associated with a 3.3-fold decline. The presence of both mutations (double mutant) resulted in a fivefold decline in transactivation activity in A172 cells. We repeated FOSL1 luciferase assay using PDX-L14 cells. We observed similar tendencies as those in A172 cells, the mut 1 STAT3 mutation resulted in a 2.7-fold decline in luciferase activity in PDX-L14 cells, while the mut 2 STAT3 mutation was associated with a 3.5-fold decline. The presence of double mutant resulted in a sixfold decline in transactivation activity in PDX-L14 cells (Fig. 3F). Clearly, the presence of intact STAT3 binding sites is essential for maximal FOSL1 promoter activity.

#### Activation of the FOSL1 promoter by STAT3 in glioma cells

Given the importance of the − 328 to − 336 and − 378 to − 386 GAS elements in the activation of FOSL1 promoter in glioma cells, we sought to examine whether cotransfection of a constitutively active mutant of STAT3 or a dominant negative mutant of STAT3 along with the FOSL1 promoter could enhance or suppress FOSL1 promoter activity. The constitutively active mutant of STAT3 has been shown to dimerize spontaneously in the absence of tyrosine phosphorylation, bind to DNA, and activate transcription of target molecules [16]. Figure 3G, H showed the effect of STAT3 on FOSL1 promoter activity in glioma cells. On average, cotransfection of either constitutively activated STAT3 (STAT3-CA) or wild-type STAT3 (WTSTAT3) resulted in a 1.88-fold and 1.65-fold increase in FOSL1 promoter activity, respectively, in A172 cells, while dominant negative STAT3 (STAT3-DN) resulted in a 1.45-fold decrease in FOSL1 promoter activity (Fig. 3G). Similarly, cotransfection of either STAT3-CA or WTSTAT3 resulted in a 1.52-fold and 1.82-fold increase in FOSL1 promoter activity, respectively, in PDX-L14 cells, while STAT3-DN resulted in a 1.62-fold decrease in FOSL1 promoter activity (Fig. 3H). The increase in FOSL1 promoter activity by STAT3-CA (1.88-fold and 1.82-fold in A172 and PDX-L14, respectively) correlates with the results of the luciferase assays performed with double STAT3 mutant constructs, which showed that site-specific mutations of the − 328 to − 336 and − 378 to − 386 GAS elements in the FOSL1 promoter decreased luciferase activity by fivefold in A172 (Fig. 3E, G) and sixfold in PDX-L14 cells (Fig. 3F, H). In combination, the results suggested that STAT3 activates the transcription of FOSL1 likely through the binding of STAT3 to the two GAS elements in the FOSL1 promoter.

#### TRPM7-induced FOSL1 transcriptional activation in gliomagenesis is mediated by STAT3

**Fig. 4:**
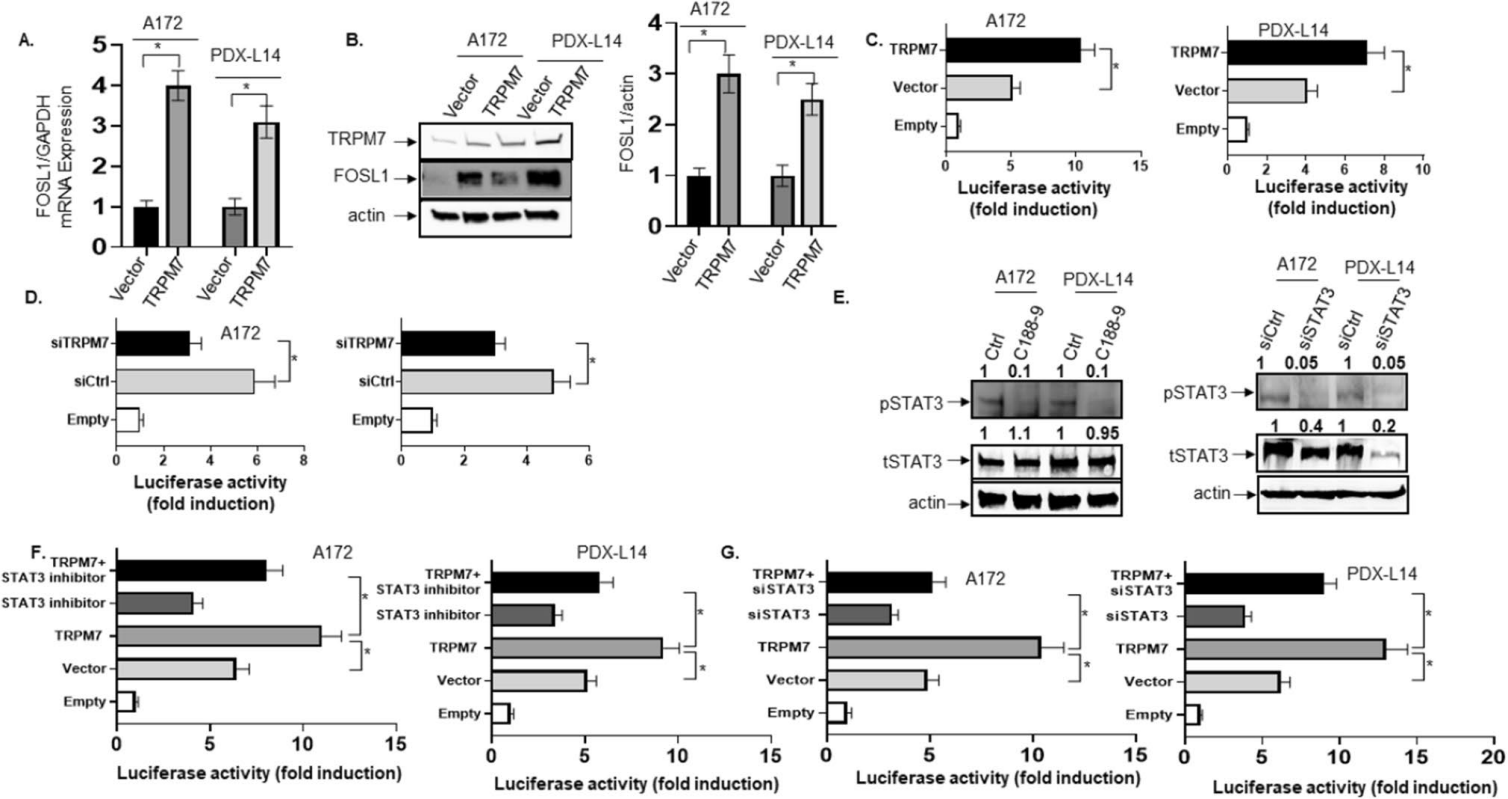
TRPM7-induced FOSL1 transcriptional activation in gliomagenesis is mediated by STAT3. **A**, **B** Upregulation of TRPM7 causes accumulation of FOSL1 mRNA (**A**) and protein (**B**). **C**, **D** TRPM7 overexpression enhances (**C**) while TRPM7 downregulation decreases (**D**) FOSL1 transcriptional activity in A172 and PDX-L14 cells. **E** The effects of STAT3 inhibitor (left) and siSTAT3 (right) on STAT3 activity. **F** STAT3 inactivation by STAT3 inhibitor prevents FOSL1 promoter activity, even in A172 (left) and PDX-L14 (right) cells overexpressing TRPM7. **G** STAT3 inactivation by siSTAT3 prevents FOSL1 promoter activity, even in A172 (left) and PDX-L14 (right) cells overexpressing TRPM7

We had previously reported that TRPM7 promotes glioblastoma proliferation and invasion through the upregulation of FOSL1 [6]. In studies using the human glioma cell lines, we found that FOSL1 mRNA (Fig. 4A) and protein (Fig. 4B) accumulate upon upregulation of TRPM7. Our previous publication showed that TRPM7 activates STAT3 through the phosphorylation of STAT3 at Tyr705 and increases glioblastoma stemenss [2]; here, we further detected the nuleocytoplasmic distribution of activated STAT3 upon expression of TRPM7. To this end, A172 and PDX-L14 cells were transfected with human wtTRPM7 for 72 h and then stained with TRPM7 and phosphorylated STAT3 antibodies, and the images were captured by Confocal microscope. As shown in Supplemental Fig. 2, TRPM7 caused an increased nuclear distribution of pSTAT3. In A172 cells, the average immunofluorescence intensity of TRPM7 and pSTAT3 in the nucleus was 43 and 55 respectively (n = 20), which was significantly stronger than those in control cells, 2.5 and 6 respectively (Supplemental Fig. 2A–C). This conclusion was confirmed in PDX-L14 cells with the average immunofluorescence intensity of TRPM7 and pSTAT3 in nucleus was 58 and 60 respectively (n = 20), which was significantly stronger than those in control cells, 3 and 5 respectively (Supplemental Fig. 2D–F). In the case of regulation between TRPM7 and FOSL1, it would be expected that overexpression of TRPM7 would enhance FOSL1 promoter activity, whereas silencing TRPM7 would reduce its activity. To ascertain if this is the case, we transiently overexpressed TRPM7 or silenced TRPM7 in A172 cells along with cotransfection of the FOSL1 promoter construct and then performed luciferase reporter assays. Results of these assays, shown in Fig. 4, demonstrated a nearly twofold and 1.74-fold increase in FOSL1 transcriptional activity in both A172 and PDX-L14 cells overexpressing TRPM7 (Fig. 4C) compared to a 1.9-fold and 1.62-fold reduction in the two cell lines underexpressing TRPM7 (Fig. 4D). To gain an insight into the molecular mechanisms associated with FOSL1 transcription in response to changes in both TRPM7 and STAT3, the experiments involving cotransfection of TRPM7 along with a FOSL1 promoter-reporter construct following the treatment with either STAT3 inhibitor XIII, C188-9 or siRNA STAT3 (siTAT3), were conducted in both A172 and PDX-L14 cells. As Fig. 4E showed, STAT3 inhibitor XIII, C188-9, effectively inhibited STAT3 activation by decreasing phosphorylated STAT3 levels (Fig. 4E left panel), while siSTAT3 significantly inhibited both phosphorylated and total STAT3 expression (Fig. 4E right panel). It was discovered that once STAT3 is inactivated by either C188-9 or siSTAT3, overexpression of TRPM7 cannot fully induce FOSL1 promoter activity in A172 (Fig. 4F left panel and Fig. 4G left panel) and PDX-L14 cells (Fig. 4F right panel and Fig. 4G right panel). This effect indicated that TRPM7-enhanced FOSL1 promoter activity is likely mediated through the binding of STAT3 to the GAS elements in the FOSL1 promoter.

#### Deacetylation of FOSL1 at the Lys-116 residue located within its DNA binding domain leads to an increase of FOSL1 transcriptional activity

**Fig. 5:**
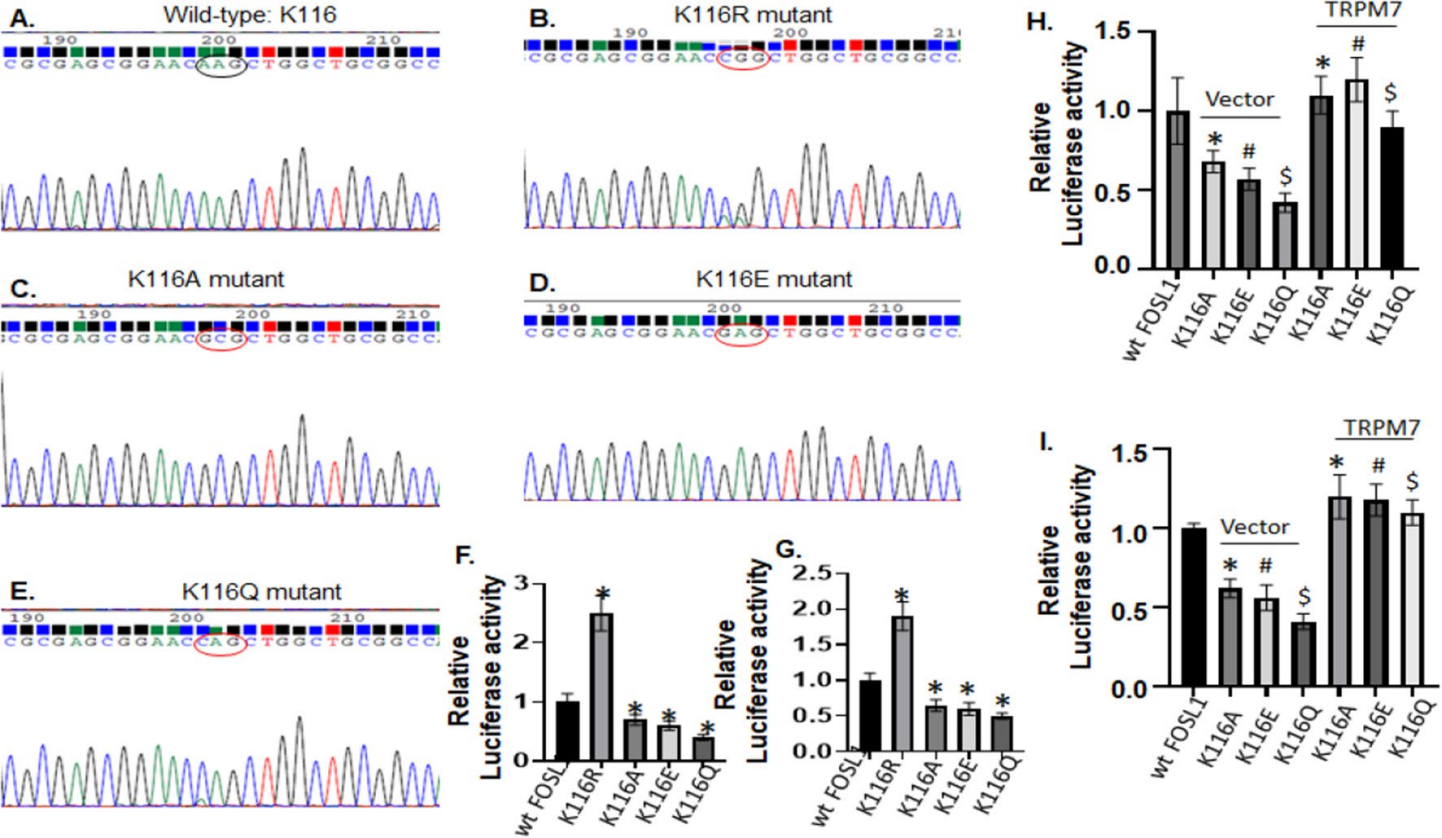
Deacetylation of FOSL1 at the Lys-116 residue increases FOSL1 transcriptional activity. **A**–**E** DNA sequencing of the DNA binding domain that contains FOSL1 acetylated lysine residue (K116, wild-type) (**A**), the deacetylation mimic FOSL1 K116R mutant (**B**), the acetylation mimic FOSL1 K116A mutant (**C**), K116E mutant (**D**), and K116Q mutant (**E**). **F**, **G** Relative luciferase activity in A172 (**F**) and PDX-L14 (**G**) cells cotransfected with wild-type and mutant FOSL1 constructs, together with FOSL1-luc. **H**, **I** Changes of relative luciferase activity in A172 (**H**) and PDX-L14 (**I**) cells transfected with acetylation mimic FOSL1 mutants caused by TRPM7

A previous report showed that IL-6/STAT3 inflammatory signaling axis induces the deacetylation of FOSL1 at the Lys-116 residue located within its DNA binding domain, leading to the increase of FOSL1 transcriptional activity and acquisition of colorectal cancer (CRC) stem-like properties (stemness) [17]. Therefore, we asked whether FOSL1 is also deacetylated during the process of transactivating FOSL1 gene by TRPM7. Due to the commercially unavailability of the acetyl-Lys 116-specific antibody against FOSL1 (K116AC), we made several FOSL1 mutants at the DNA binding domain that contains FOSL1 acetylated lysine residue (K116), including the deacetylation mimic K116R mutant, and three acetylation mimic K116A, K116E, and K116Q mutants (Fig. 5A–E). The glioma A172 and PDX-L14 cells were cotransfected with wild-type and mutant FOSL1 constructs at Lys116, together with FOSL1-luc and pRL-TK as an internal control reporter. As shown in Fig. 5, the FOSL1 K116R mutant that mimics deacetylation at the Lys-116 residue resulted in an increase of FOSL1-luc activity in A172 (2.5-fold increase, Fig. 5F) and PDX-L14 (1.9-fold increase, Fig. 5G), whereas the acetylation mimic FOSL1 K116/A/E/Q mutants were characterized by an decrement of FOSL1 reporter luciferase activity in A172 (30–60% decrease, Fig. 5H) and PDX-L14 (35–50% decrease, Fig. 5I). To examine the effects of TRPM7 on deacetylation of FOSL1 during the process of transactivating the FOSL1 gene, we cotransfected TRPM7 construct, together with acetylation mimic K116A, K116E, and K116Q mutants and FOSL-luc into A172 and PDX-L14, followed by measuring the FOSL1 luciferase activity. As shown in Fig. 5H, I, TRPM7 led to an increment of FOSL1 reporter luciferase activity in both A172 (Fig. 5H) and PDX-L14 (Fig. 5I) cells. These mutagenesis and luciferase reporter assays provided experimental evidence that deacetylation of FOSL1 at the Lys-116 residue resulted in an increase of FOSL1 transcriptional activity.

### ChIP-qPCR analysis demonstrates that STAT3 can directly bind to the FOSL1 promoter in A172 cells

**Fig. 6:**
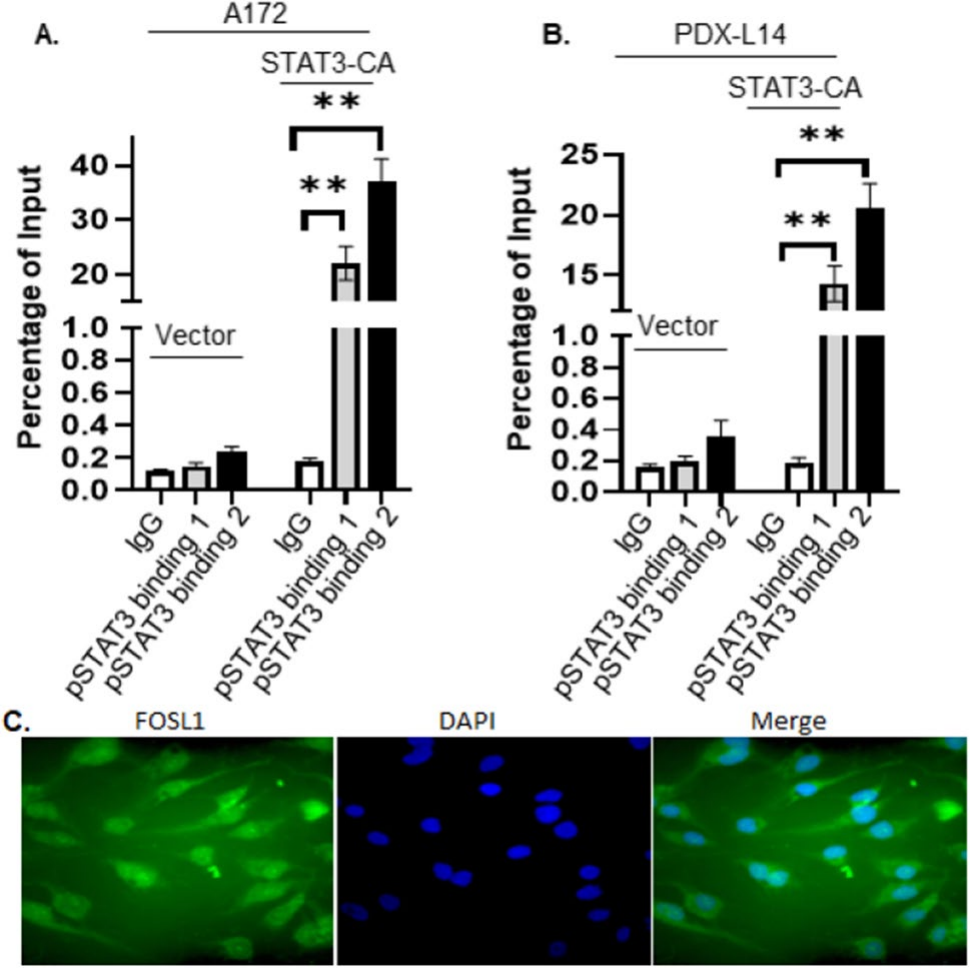
ChIP-qPCR analysis of STAT3 direct binding to the FOSL1 promoter. ChIP-qPCR results were analyzed by evaluating signal of enrichment over noise normalized to input. A172 cells (**A**) and PDX-L14 cells (**B**) were transduced with STAT3-CA and vector. DNA levels were normalized to the relative inputs (n = 3 independent experiments; **p < 0.001 among groups by one-way ANOVA). The representative nuclear staining of FOSL1 in A172 cells was shown in **C**, magnification ×40

We then investigate whether STAT3 present in nuclear lysate of glioma cells could bind to the − 328 to − 336 and − 378 to − 386 at the FOSL1 promoter by performing ChIP-qPCR. A172 cells (Fig. 6A) and PDX-L14 cells (Fig. 6B) transduced with STAT3-CA and vector were crosslinked with 1% formaldehyde and underwent Micrococcal Nuclease digestion (MNase Digestion). The chromatin fragments generated (0.2–1 kb) were then incubated with 5 μg of polyclonal anti-phosphorylated STAT3 or normal rabbit IgG (negative control). Antibody-bound protein/DNA complexes were eluted and subjected to qPCR. An aliquot of chromatin that was not incubated with an antibody was used as the input control sample (10% total input sample). qPCR was performed using primers designed to surround the − 328 to − 336 and − 378 to − 386 binding sites of the FOSL1 promoter using the Primer 3 tool (https://primer3.ut.ee/). The primer sequences for each site are listed in Table 1. Data were analyzed using the percent input method and using normal rabbit IgG as negative controls. The ChIP experiments were performed in triplicates, and the results were presented together with the background signal and standard error. Interestingly, we found that pSTAT3 co-immunoprecipiated more fragments of the FOSL1 promoters (21.99% in binding 1 and 37.2% in binding 2) than the empty vector (0.18%) in A172 cells (Fig. 6A; Table 2). A similar trend was observed in PDX-L14 cells as well (14.29% in binding 1 and 20.54% in binding 2 vs 0.19% in vector, Fig. 6B; Table 3). These results demonstrated that STAT3 bound directly to the FOSL1 promoter region at the level of STAT3 putative binding sites and suggested that it potentially served as FOSL1 transcription regulator. As shown in Fig. 6C, FOSL1 was localized in the nucleus of A172 glioma cells.

**Table 2:** Calculation of percent input of A172

A172					
**Vector**					
		Step 1			Step 2
		*Adjusted input to 10%			Percent input
	Raw Ct	(Ct Inpu-3.32)		Triplicate average Ct	100*2^ (Adjusted input-Ct(IP)
Input (10%)	28.35	25.03	Adjusted input	25.03	
			Negative (IgG)	34.69	0.12
			pSTAT3 antibody binding 1	34.46	0.15
			pSTAT3 antibody binding 2	33.72	0.24
**STAT3-CA**					
		Step 1			Step 2
		*Adjusted input to 10%			Percent input
	Raw Ct	(Ct Inpu-3.32)		Triplicate average Ct	100*2^ (Adjusted input-Ct(IP)
Input (10%)	28.35	25.03	Adjusted input	25.03	
			Negative (IgG)	34.19	0.18
			pSTAT3 antibody binding 1	27.22	21.99
			pSTAT3 antibody binding 2	26.46	37.20

**Table 3:** Calculation of percent input of PDX-L14

PDX-L14					
**Vector**					
		Step 1			Step 2
		*Adjusted input to 10%			Percent input
	Raw Ct	(Ct Inpu-3.32)		Triplicate average Ct	100*2^ (Adjusted input-Ct(IP)
Input (10%)	28.35	25.03	Adjusted input	26.07	
			Negative (IgG)	35.32	0.16
			pSTAT3 antibody binding 1	35.02	0.20
			pSTAT3 antibody binding 2	34.20	0.36
**STAT3-CA**					
		Step 1			Step 2
		*Adjusted input to 10%			Percent input
	Raw Ct	(Ct Inpu-3.32)		Triplicate average Ct	100*2^ (Adjusted input-Ct(IP)
Input (10%)	28.35	25.03	Adjusted input	26.07	
			Negative (IgG)	35.11	0.19
			pSTAT3 antibody binding 1	28.87	14.29
			pSTAT3 antibody binding 2	28.35	20.54

### The expression of TRPM7, ALDH1, and FOSL1 protein is associated with grades of glioma in glioma patients

The protein expression of TRPM7 and GSC markers ALDH1 and FOSL1 were then examined by IHC in glioma brain TMA obtained from glioma patients at BioCoreUSA. TRPM7 and ALDH1 proteins were expressed in grade II astrocytoma, grade III astrocytoma, grade IV GBM, and normal tissues, which were localized in both cytoplasm and nucleus.

#### TRPM7

**Fig. 7:**
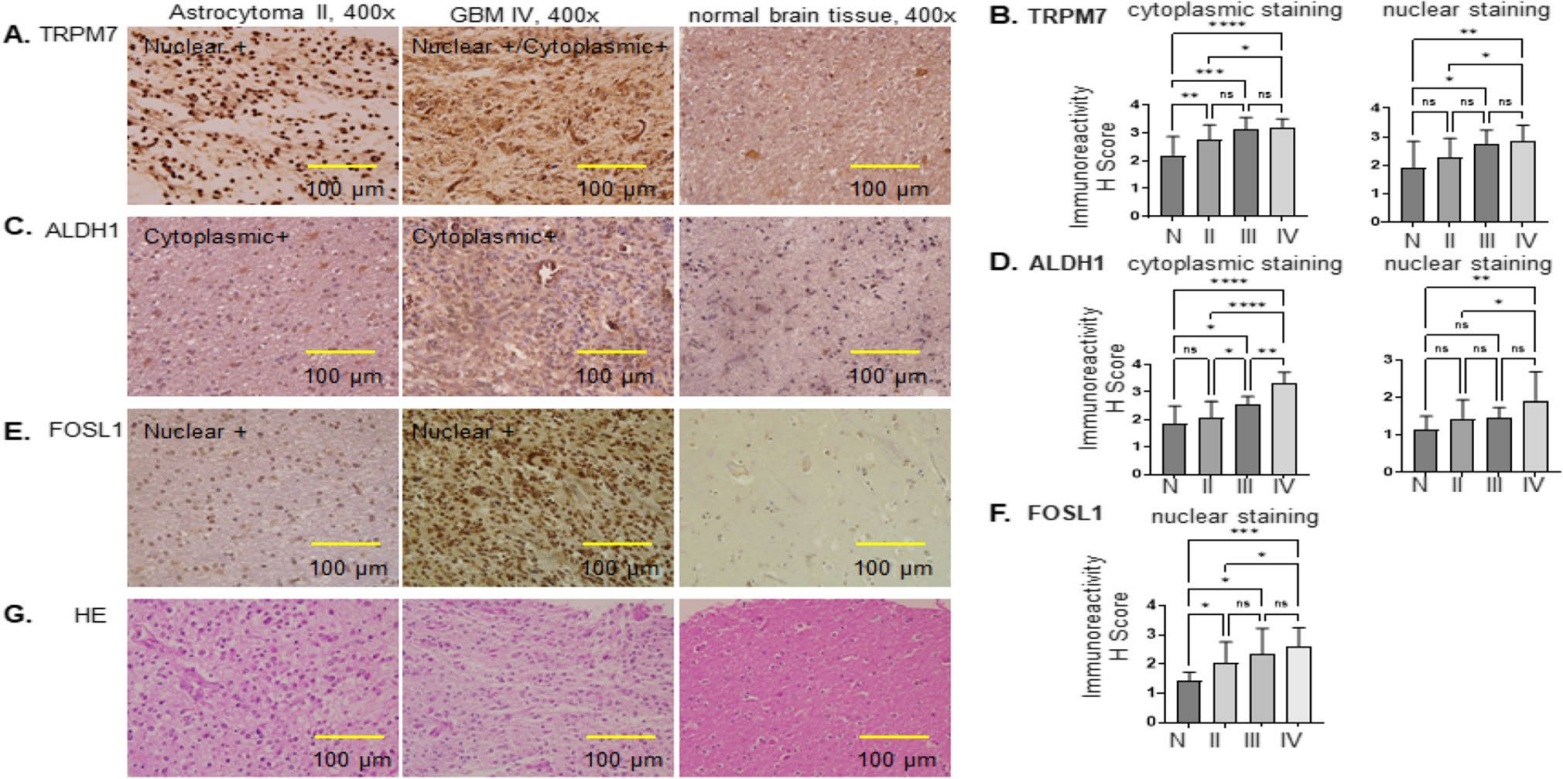
TRPM7 protein expression is associated with grade and correlates to ALDH1 and FOSL1 in glioma patients. **A** TRPM7 protein was expressed in grade II gliomas (left), grade IV GBM (middle), and normal brain tissue (right). **B** Quantification of IHC results in grade II, grade III, and grade IV GBM compared with that of normal brain tissue, as well as IHC results compared among different grades of gliomas. **C** ALDH1 protein was expressed in grade II gliomas (left), grade IV GBM (middle), and normal brain tissue (right). **D** Quantification of IHC results in grade II, grade III, and grade IV GBM compared with that of normal brain tissue, as well as IHC results compared among different grades of gliomas. **E** FOSL1 protein was expressed in grade II gliomas (left), grade IV GBM (middle), and normal brain tissue (right). **F** Quantification of IHC results in grade II, grade III, and grade IV GBM compared with that of normal brain tissue, as well as IHC results compared among different grades gliomas. **G** HE staining in grade II (left), grade IV (GBM), and normal brain tissue (right). All images were captured at a magnification of ×40. Statistical significance was determined by one-way ANOVA test. *p < 0.05, ** p < 0.01, ***p < 0.001, ****p < 0.0001. *HE* hematoxylin and esosin

Figure 7A showed the representative staining of TRPM7 protein in grade II astrocytoma (Fig. 7A left, nuclear staining), grade IV GBM (Fig. 7A middle, both cytoplasm and nuclear staining) and normal brain tissues (Fig. 7A right). Quantification of the IHC results revealed that positive cytoplasmic staining of TRPM7 was significantly higher in grade II (n = 51, p < 0.01), grade III (n = 12, p < 0.001), grade IV GBM (n = 12, p < 0.0001) compared with that of normal brain tissue (n = 10) (Fig. 7B left); positive nuclear staining of TRPM7 in grade III gliomas (p < 0.05) and grade IV (p < 0.01) was significantly higher than that of normal brain tissue (Fig. 7B right). In terms of different grades of glioma, cytoplasmic staining of TRPM7 was significantly increased in grade IV GBM compared with that in grade II glioma (p < 0.05) (Fig. 7B left), while nuclear staining of TRPM7 was significantly increased in grade IV GBM compared wth that of grade II gliomas (p < 0.05) (Fig. 7B right). The positive association between increased TRPM7 protein expression and glioma grades strongly indicated that the TRPM7 protein can be used as a diagnostic marker and potential drug target for glioma patients.

#### ALDH1

Figure 7C showed the representative staining of ALDH1 protein in grade II astrocytoma (Fig. 7C left, cytoplasmic staining), grade IV GBM (Fig. 7C middle, cytoplasm), and normal brain tissues (Fig. 7C right). Quantification of IHC results revealed that positive cytoplasmic staining of ALDH1 was significantly higher in grade III (n = 12, p < 0.05), grade IV GBM (n = 12, p < 0.0001) compared with that of normal brain tissue (n = 10) (Fig. 7D left); positive nuclear staining of ALDH1 in grade IV GBM (p < 0.01) was significantly higher than that of normal brain tissue (Fig. 7D right). In terms of different grades of glioma, cytoplasmic staining of ALDH1 was significantly increased in grade IV GBM compared with that in grade II glioma (p < 0.0001) and grade III astrocytoma (p < 0.01) (Fig. 7D left), while nuclear staining of ALDH1 was significantly increased in grade IV GBM compared with that of grade II gliomas (p < 0.05) (Fig. 7D right). The positive association between increased ALDH1 protein expression and glioma grades further confirmed the well-established role of ALDH1 protein as a prognostic marker in glioma patients.

#### FOSL1

FOSL1 protein was mainly expressed in the nucleus. Figure 7E showed the representative nuclear staining in grade II astrocytotoma (Fig. 7E left) and grade IV GBM (Fig. 7E middle), as well as very weak nuclear staining in normal brain tissues (Fig. 7E right). Quantification of IHC results revealed that positive nuclear staining of FOSL1 was significantly higher in grade II (n = 51, p < 0.05), grade III (n = 12, p < 0.05), grade IV GBM (n = 12, p < 0.0001) compared with that of normal brain tissue (n = 10) (Fig. 7F). The positive association between increased FOSL1 protein expression and glioma grades strongly implicated the FOSL1 protein as a diagnostic marker and a potential drug target for glioma patients.

### TRPM7 protein expression correlates to the expression of ALDH1 and FOSL1 in glioma patients

**Fig. 8:**
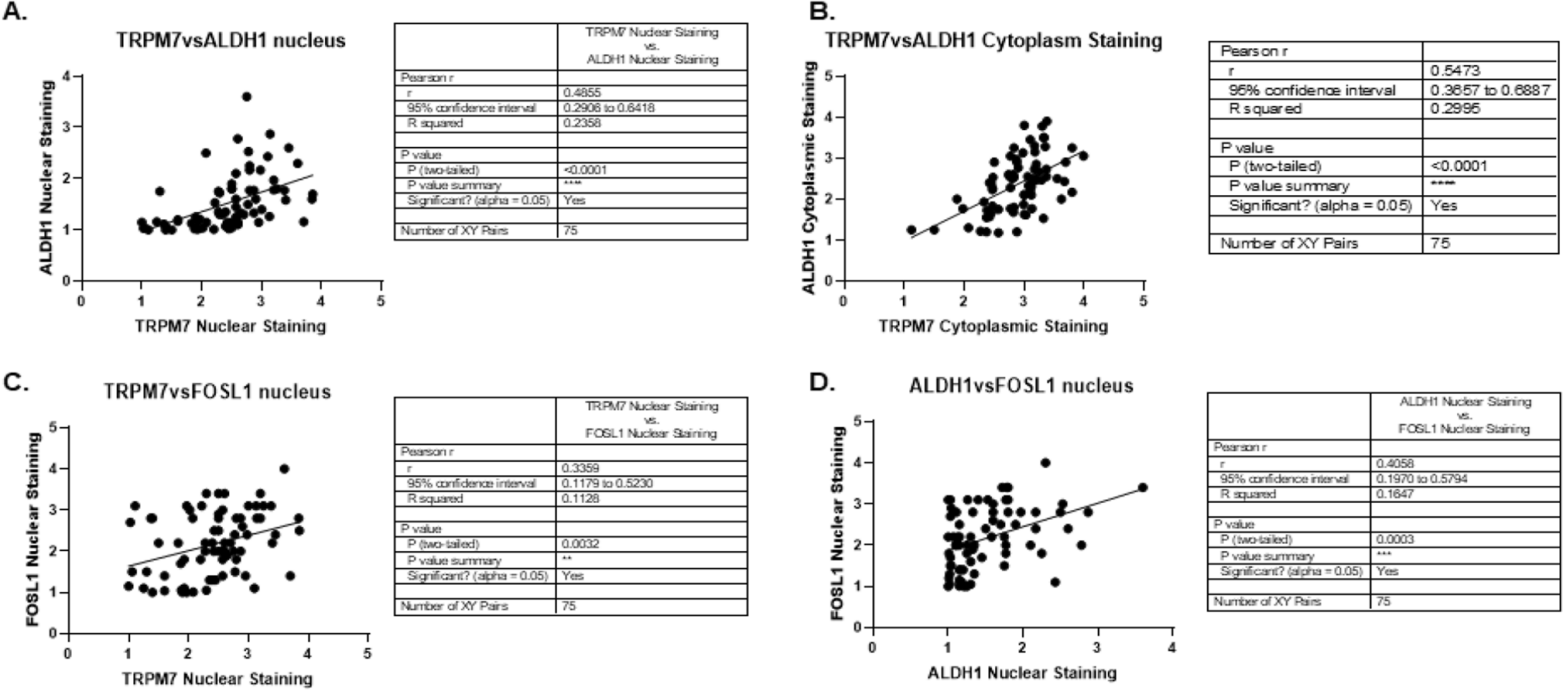
TRPM7 protein expression correlates to the expression of ALDH1 and FOSL1 in glioma patients. Pearson’s correlation curve revealed the positive relationship between the nuclear staining of TRPM7 and ALDH1 (**A**), the cytoplasmic staining of TRPM7 and ALDH1 (**B**), the nuclear staining of TRPM7 and FOSL1 (**C**), and nuclear staining of ALDH1 and FOSL1 (**D**)

Next, by quantification of TRPM7 protein expression and ALDH1 protein expression followed by Pearson’s correlation analysis, we found a significant positive correlation not only between the nuclear staining of TRPM7 and ALDH1 (r = 0.4855, p < 0.0001) (Fig. 8A) but also cytoplasmic staining (r = 0.5473, p < 0.0001) (Fig. 8B). Interestingly, the positive correlation was also found between nuclear staining of TRPM7 and FOSL1 (r = 0.3359, p = 0.0032) (Fig. 8C). In addition, we observed the significant positive correlation between nuclear staining of ALDH1 and FOSL1 (Fig. 8D). These results indicated that both TRPM7 and FOSL1, similar to the established GSC marker ALDH1, have the potential to serve as diagnostic markers and potential drug targets in glioma patients.

## Discussion

In the current study, we investigated the role of TRPM7/STAT3/FOSL1 axis in promoting glioma stemness and gliomagenesis. The major findings include: (1) FOSL1 downregulation suppresses glioma stemness/tumor cell growth and decreases the expression of GSC markers CD133 and ALDH1; (2) mechanistically, TRPM7 induces STAT3-mediated FOSL1 transcriptional activation during gliomagenesis; (3) clinico-pathologically, the TRPM7 protein expression correlates to the protein expression of ALDH1 and FOSL1 and is associated with grades of malignant glioma. In this study, we selected the two glioma cell lines A172 and U87MG, as well as patient-derived xenoline PDX-L14 based on their different molecular features. Both A172 and U87MG harbor PTEN and CDKN2A (p16^INK4a^) mutations [18–20] and are reported as isocitrate dehydrogenase (IDH) wild type [21], while U87MG has CDKN2C (p18^INK4c^) mutation [21] and O6-methylguanine-DNA methyltransferase (MGMT) promoter methylation [22]. In general, the U87MG cell line serves as a model for the mesenchymal subtype as it has neurofibromin 1 (NF1) deletion and wild-type expression of epidermal growth factor receptor (EGFR), while the A172 cell line with EGFR expressed serves as a model similar to the neural subtype [23]; although in 2017, this subtype was suggested to have arose from contamination of the original samples with nontumor cells [24, 25]. PDX closely mimics the biological and physiological features of in vivo real cells and tissues; therefore, we used the PDX-L14 (a proneural subtype) to validate further the conclusions drawn from the two glioma cell lines, A172 and U87MG. Due to high inter tumor and intra-tumor heterogeneity in GBM, each subtype has different sphere-forming capacity as we showed in Fig. 1 and Supplemental Fig. 1.

FOSL1 is known to be a component of AP1 transcription factor complexes, and whether CD133 and ALDH1 are FOSL1's direct or indirect transcriptional targets is beyond the scope of this manuscript. However, we located the CD133 and ALDH1 promoter sequences through Ensembl Genome Browser, then used a virtual laboratory PROMO to identify putative transcription factor binding sites (TFBS) in the promoter DNA sequences of CD133 and ALDH1. We found three predicted binding sites of AP1 to CD133 gene promoter at nucleotides 267–273, 558–568, and 859–865, and three to ALDH1 gene promoter at 414–421, 539–545, and 744–750. In the future, we will develop ChIP assay to provide further evidence of the direct binding of AP1 to promoter regions of CD133 and ALDH1.

The nuclear oncoprotein FOSL1 is overexpressed in most solid tumors including lung cancer [26], breast cancer [27], ovarian cancer [28], prostate cancer [29], gastric cancer [30], colorectal cancer (CRC) [31], head and neck squamous cell carcinomas [32], and GBM [33], and has emerged as a prominent therapeutic target [34]. FOSL1 protein is localized in the nucleus and cytoplasm [35] and plays an essential role in cancer cell proliferation, invasion/metastasis [6], epithelial-to-mesenchymal transition (EMT) [31], and antitumor immunity [36]. The regulation of FOSL1 gene expression is multifaceted. FOSL1 is regulated at both transcriptional and post-translational levels, such as phosphorylation and deacetylation [35]. In CRC, basal activity of extracellular signal-regulated kinase (ERK) is required to induce transcription of the FOSL1 gene, while additional higher levels of ERK activity stabilize FOSL1 against proteasome-dependent degradation [37]. IL-6/STAT3 inflammatory signaling axis induces the deacetylation of FOSL1 at the Lys-116 residue located within its DNA binding domain, leading to the increase of FOSL1 transcriptional activity and acquisition of CRC stem-like properties (stemness) [17]. The mutagenesis and luciferase reporter assays in our study (Fig. 5) provided the experimental confirmation that deacetylation of FOSL1 at the Lys-116 residue resulted in an increase in FOSL1 transcriptional activity. We infer that increment of FOSL1 reporter luciferase activaty is partly caused by TRPM7-induced deacetylation of FOSL1 at Lys-116, in addition to TRPM7-induced activation of STAT3. However, due to the unavailability of acetyl-lysine 116-specific antibody against FOSL1 (K116AC), we are currently unable to run Western blot to confirm the inference. On other hand, the increased transcriptional activity of FOSL1 by STAT3 upregulates EMT-promoting factors (ZEB1, Snail, Slug, MMP-2, and MMP-9) in CRC [31]. In transformed thyroid cells, the Ras-dependent PI3K triggered the transactivation of FOSL1, while the constitutive activation of FOSL1 required the additional activation of the MEK/ERK pathway by phosphorylation [38]. The Wnt/β-catenin signaling activated the FOSL1 transcription and drove EMT of glioma cells [39]. In our study, we are the first to report that in glioma, the FOSL1 induction occurred at the transcriptional level in a manner dependent on STAT3, during which phosphorylated post-translational modification was required for STAT3 activation to directly bind to the FOSL1 promoter (Fig. 6, ChIP).

STAT3 is widely studied in malignancy during the last vicennial as it regulates networks of genes involved in oncogenesis [16], cell proliferation [2, 40], cell cycle progression [40], angiogenesis [41], metastasis, and evasion of apoptosis [42]. An emrging evidence showed that hyperactivation of STAT3 promotes stem cell-like trait in many types of cancers. It is encouraging and inspiring that during the past decade, more knowledge is gained on how STAT3 is regulated in cancer stem cells and the mechanisms by which STAT3 contributes to poor prognosis in aggressive cancer [43]. Through activating crucial genes related to breast cancer stem cells, STAT3 contributes to breast cancer metastasis and therapeutic resistance [44]. For instance, the activated STAT3 enriches the expression of CD44 [43], physically interacts with CD44 and NF-κB, activates the telomerase (hTERT), promotes a cancer stem phenotype [43]. Moreover, constitutive activation of STAT3 modulates NF-κB signaling and enhances liver cancer stemness [45]. Simultaneous activation of STAT3 and NF-κB signaling modulates Notch-related genes in GSC [46]. Since a major cause of chemoresistance is cancer stemness, it is as expected that canonical STAT3 signal transduction pathways play an essential role in cancer stem cell-associated chemoresistance. STAT3 activates downstream EMT-inducing genes zinc finger protein Snail [47–49] and Slug [48], Twist [50, 51], zinc finger E-box binding homeobox 1 (ZEB1) [52, 53], which in turn augment the function of ATP-binding cassette (ABC) membrane transporters such as multidrug resistance mutation 1 (MDR-1), ABC sub-family G member 2/3 (ABCG2/3), and ABC sub-family C member 2/4/5 (ABCC2/4/5). In our study, we found that STAT3 regulates downstream gene target, FOSL1, which is closely related to glioma stemness and therefore facilitates gliomagenesis.

FOSL1 gene, mapped in the 11q13, has been found to be a potential diagnostic marker and drug target for a variety of cancers. Zhang et al. reported that FOSL1 gene expression increases both at the mRNA and protein levels in oral squamous cell carcinoma (OSCC). Both nuclear and cytoplasmic FOSL1 protein expression significantly increased in the OSCC cancerous tissues than those in the paired adjacent non-malignant epithelia. Furthermore, increased nuclear FOSL1 expression was correlated with lymph node metastasis [54]. Increased copy number and mRNA overexpression of FOSL1 gene are frequently observed in primary breast cancers, independently and irrespectively of the patients’ lymph node axillary metastatic status [55]. Analysis from transcriptomic FPKM expression data obtained from UCSC Xeba website (https://xenabrower.net/) by Jin’s group demonstrated that FOSL1 can better predict glioma prognosis [56]. Our previous bioinformatic analysis from TCGA mRNA data showed that FOSL1 serves as a diagnostic and prognostic marker for glioma patients [6]. In the current study, we further detected FOSL1 protein expression using glioma patients brain tissues. In addition, we previously reported that TRPM7 enhances GSC stemness by regulating the established GSC marker ALDH1 and promotes the induction of ALDH1 activity in glioma cells [2]; therefore, we further tested the relationship between FOSL1 and GSC stemness markers TRPM7 and ALDH1 by IHC assay. We found FOSL1 levels were positively correlated with the glioma grades in a total of 75 glioma patients (Fig. 7) and correlated to the established GSC markers ALDH1 and TRPM7 (Fig. 8), suggesting FOSL1 is a diagnostic marker and potential therapeutic target for glioma patients. In all, the current data conform with our previous findings which highlight FOSL1’s potential predictive/prognostic role for glioma patients.

Our previous studies have delineated several TRPM7-mediated pathways contributing to the gliomagenesis and glioma stemness. One of our findings shows that TRPM7 activates JAK2/STAT3 signaling pathways and leads to increased glioma cell proliferation and migration/invasion [2]. Another finding demonstrates that TRPM7 regulates the upregulation of FOSL1 oncogene through non-coding RNA and consequently results in induction of gliomagenesis [6]. These interactions prompted us to investigate the mechanism by which TRPM7 modulates FOSL1 transcriptional activation in glioma cell. In the current study, we found a new pathway that TRPM7 transactivates the FOSL1 gene through transcription factor STAT3 and enhances glioma stemness.

Glioblastoma is the most frequent and devastating form of adult primary brain tumor [57]. Intratumoral heterogeneity is the major challenge in the treatment of glioblastoma [58]. GSCs have capacity of clonogenic self-renewal and asymmetric cell division, which contribute to glioblastoma’s rapid progression and invasion [59], treatment resistance, and recurrence [60]. The presence of GSCs and stochastic state transition between different molecular subtypes/subpopulations [61], as well as the conversion of differentiated cancer cells (non-stem states) into GSCs after primary chemotherapy contribute to the complexity of heterogeneity [61, 62]. Heterogenenous GSCs, which is described as multiple GSC subtypes/subpopulations coexisting within a single tumor, is a major reason that multiple different pattern of signaling pathway are activated in GSCs, such as PI3K/AKT, MEK/ERK, JAK/STAT, WNT/β-catenin, NF-κB, and MAPK/p38 [60, 63]. Our present study discovered a novel signaling pathway of TRPM7/STAT3/FOSL1 axis that leads to glioma aggressiveness through stemness, suggesting novel therapeutic opportunities for the malignant disease.

The therapeutic goal and solution for glioma is to target distinct subpopulations of GSCs that may simultaneously reside within a single tumor and within possible multiple signaling pathways. In addition, due to the conversion among different glioma subpopulations, and the transition between non-stem and stem-like states, treatments to tackle the dynamic processes instead of targeting only a small subpopulation of GSCs need to be considered in the future.

## Conclusion

This study illustrated the important role of FOSL1 in self-maintenance of GSCs, and its potential as a GSC marker. The findings deciphered the detailed molecular mechanism underlying TRPM7 transactivated FOSL1, which was mediated via STAT3. Our findings ffer future directions for successful GBM therapy.

### Supplementary Information

Below is the link to the electronic supplementary material.

Supplementary file1 (DOCX 1207 KB)

## Data Availability

Data generated during the study are subject to a data sharing mandate and available in a public repository that does not issue datasets with DOIs.
