# Supplementary material for: TRPM7 transactivates the FOSL1 gene through STAT3 and enhances glioma stemness
Source: Cell Mol Life Sci. 2023 Aug 29;80(9):270. doi: 10.1007/s00018-023-04921-6 (PMC10465393; doi:10.1007/s00018-023-04921-6)
Supplement: Supplementary file 1 — Supplementary file1 (DOCX 1207 KB) [file 18_2023_4921_MOESM1_ESM.docx]

**Supplemental** **Figures**

 Supplemental Fig 2. TRPM7 causes the nuclear translocation of STAT3. (A-C). A172 cells transfected with wtTRPM7 for 72 h were stained with TRPM7 (A) and pSTAT3 (B), Magnification 63x. (C). Quantitation of nuclear TRPM7 and nuclear phosphorylated STAT3 staining. (D-F). PDX-L14 cells transfected with wtTRPM7 for 72 h and stained with TRPM7 (D) and pSTAT3 (E), Magnification 63x. (F). Quantitation of nuclear TRPM7 and nuclear phosphorylated STAT3 staining.

Supplemental Fig 1. FOSL1 inhibitor SR11302 impairs stemness *in vitro*. (A) Significantly decreased expression of FOSL1 were confirmed in both GSCs enriched from U87MG and PDX-L14 cells treated with 10µM SR11302. (B) Representative limiting dilution experiments on U87MG GSCs treated with SR11302 and controls. Bar plot shows the estimated stem cell frequency with the confidence interval; chi-square p<0.001. (C) Representative limiting dilution experiments on PDX-L14 GSCs treated with SR11302 and controls. Bar plot shows the estimated stem cell frequency with the confidence interval; chi-square p<0.05.
